# *BJC* moves forward

**DOI:** 10.1038/sj.bjc.6600140

**Published:** 2002-01-07

**Authors:** R A Weiss

## Abstract

*British Journal of Cancer* (2002) **86**, 1–1. DOI: 10.1038/sj/bjc/6600140 www.bjcancer.com

© 2002 The Cancer Research Campaign

## 

The *British Journal of Cancer* wishes all readers and contributors a happy and productive New Year. *BJC* continues to broaden its range and strengthen its international base as a high ranking journal in cancer research. It encompasses clinical, epidemiological and experimental research. Our relatively new section on Genetics and Genomics is growing and *BJC* as a whole has increased its impact rating and high quality. We are expanding our editorial comment and we are pleased that readers particularly value the concise and incisive short reviews on the latest advances in cancer research.

Starting with this issue, we are pleased to announce a new publisher, Nature Publishing Group. NPG will provide a dynamic publishing environment in which *BJC* has a pivotal place among NPG's cancer journals. Nature's news services and Nature Cancer Update will facilitate access to numerous developments in cancer research as well as ease of cross-referral. We look forward to rapid development of on-line submission and processing of research papers and to increasing our editorial and review content. Improved production times and on-line availability will benefit authors and readers alike.

In February the owner of *BJC*, The Cancer Research Campaign, will merge with the Imperial Cancer Research Fund to form ‘Cancer Research UK’. The new organization will be the world's largest cancer research charity, combining complementary strengths to advance the treatment of cancer patients and, no less importantly, to understand the nature and causes of cancer, and to prevent it. From this exciting development *BJC* will gain strength while maintaining its independent outward looking, international scope and reputation for novelty combined with reliability.

Robin A Weiss

*Editor-in-Chief*

